# Prevalence and Genomic Features of Aichivirus D in Yaks in Western China

**DOI:** 10.3390/ani16182902

**Published:** 2026-09-15

**Authors:** Yang Su, Musha Jili, Guangfu Zhao, Qibing Gu, Falong Yang, Hui Zhang, Huanrong Zhang, Dechun Chen, Yutao Shi, Mingjing Gu, Kebu Munai, Wangqing Banma, Kehamo Abi

**Affiliations:** 1 Key Laboratory of Veterinary Medicine of Universities in Sichuan, College of Animal & Veterinary Sciences, Southwest Minzu University, Chengdu 610041, China; 2 Animal Disease Prevention and Control Center of Aba Prefecture, Barkam 624000, China

**Keywords:** Aichivirus D, yak, genotype, recombination, molecular characteristics

## Abstract

Aichivirus D (AiV-D), a member of the genus Kobuvirus within the family Picornaviridae, is an emerging pathogen associated with diarrhea in multiple livestock species, including cattle, dairy cattle, Tibetan sheep, and yaks. In this study, a total of 280 fecal samples were collected from yaks with diarrhea in Sichuan, Gansu, and Qinghai Provinces, western China, between June 2025 and February 2026. The overall positive rate of AiV-D was 30.00% (84/280), and the farm-level positive rate was 87.5% (7/8). Four complete yak AiV-D genomes (GenBank: PZ424445–PZ424448) were obtained from the positive samples, with genome sizes ranging from 8369 to 8475 nt and GC contents ranging from 55.66 to 55.85%. Each genome contained a single open reading frame (ORF) of 7509–7518 bp, encoding a typical picornaviral polyprotein consisting of a leader protein (L), structural proteins P1 (VP0, VP3, and VP1), and nonstructural proteins P2 (2A, 2B, and 2C) and P3 (3A, 3B, 3C, and 3D). Pairwise comparisons of the ORF revealed nucleotide and amino acid identities of 82.07–99.63% and 81.96–99.56%, respectively, among the four strains. Phylogenetic analysis based on the complete genomes revealed that strain BKV/YAK/FX1/2025/CHN clustered with the bovine reference strain BKV5 (84.70% nt/88.35% aa identity), whereas the other three strains grouped with the yak reference strain BKV/Yak/SCDF77/2021/CHN (GenBank: OP776109.1) (89.80–93.07% nt 95.53–96.84% aa identity). Recombination analysis predicted a recombination event in strain BKV/YAK/SF1JK6/2025/CHN, with breakpoints located in the nonstructural protein coding region (2C–3D). Additionally, eight full-length VP0, VP3, and VP1 gene sequences were amplified from the AiV-D-positive samples, and a few co-mutational amino acid substitutions were identified in these structural proteins. Phylogenetic analysis based on the VP1 gene further divided the eight strains into two distinct clades: six strains formed an independent clade and were designated as a novel sub-genotype within AiV-D4, tentatively named AiV-D4-L1, closely related to but clearly distinguishable from the AiV-D4 reference strains reported in China, while the remaining two strains (BKV/YAK/FX1/2025/CHN and BKV/YAK/FX77/2025/CHN) clustered with the bovine reference strain and were designated as a novel genotype, AiV-D5. These results reveal genetic diversity among yak-derived AiV-D strains and suggest possible host adaptability or cross-host transmission, warranting further investigation in future studies. However, as all the samples were collected from diarrheic yaks without a healthy control group, the potential association between AiV-D and diarrhea requires further validation.

## Simple Summary

Yaks are an important livestock species on the Qinghai–Tibet Plateau and provide livelihoods for local herders. However, diarrhea is a common health issue in yak calves, leading to economic losses. In this study, we investigated the presence and genetic characteristics of Aichivirus D (AiV-D), a newly recognized virus, in diarrheic yaks from three provinces in western China. We collected 280 fecal samples and reported that 30% of them tested positive for AiV-D, and the virus was present on nearly all the farms surveyed. We obtained the complete genetic sequences of four viral strains and identified a novel genotype (AiV-D5) and a novel sub-genotype within AiV-D4 (AiV-D4-L1). Importantly, we discovered that AiV-D is highly genetically diverse and may undergo recombination, a process that can generate new viral variants. These findings suggest that AiV-D is widespread in yak populations and that recombination may help the virus adapt and evolve. Our results provide useful information for monitoring viral diarrhea in yaks and suggest a potential role for AiV-D under these conditions, which is important for maintaining the health and productivity of these animals.

## 1. Introduction

Kobuvirus (KoV), a member of the picornavirus family, is a pathogen associated with diarrhea in humans and various animals worldwide [1]. First identified in 1989 in fecal samples from patients with acute gastroenteritis in Aichi Prefecture, Japan, it was named Aichivirus [2]. The complete Aichivirus genome consists of a 5′-UTR, a large open reading frame (ORF) and a 3′-UTR, with the ORF encoding structural protein P1 (VP0, VP3, and VP1) and the nonstructural proteins P2 (2A–2C) and P3 (3A–3D) [3]. The International Committee on Taxonomy of Viruses (ICTV) has classified aichiviruses into six genera, named Aichivirus A-F. Aichivirus A primarily infects humans but has also been detected in dogs, cats, and birds [2,4,5,6]; Aichivirus B has been detected in cattle, sheep, and ferrets [7,8,9]; Aichivirus C has been detected in pigs and sheep [10,11]; Aichivirus D has been detected in cattle, Tibetan sheep, yaks, and dairy cattle [12,13,14,15]; and Aichivirus E and Aichivirus F have been detected in rabbits and bats, respectively [16,17].

AiV-D was first identified in cattle in Japan in 2015 [12] and has since been detected in Tibetan sheep [13], yaks [14], and dairy cattle [15], suggesting a possible cross-species transmission trajectory. The marked genetic diversity observed within kobuviruses is largely driven by high mutation rates and frequent recombination events [18,19]. Indeed, recombination has been documented across multiple hosts: in Tibetan sheep, intragenus recombination occurred in the 2C–3D region, with yak-origin AiV-D as the major parent and ovine Aichivirus C as the minor parent [13]; in cattle, intraspecies recombination was identified at the VP1-2A junction, involving European bovine AiV-D as the major parent and Tibetan sheep AiV-D as the minor parent [20]; in goats, cross-host recombination spanned the P1 and P3 regions, with Tibetan sheep AiV-D as the major parent and cattle AiV-D as the minor parent [21]; and in porcine kobuvirus, intraspecies recombination events have been reported in the VP1, 2C, and 3D regions among strains from distinct geographic origins [18,19]. Collectively, these findings suggest that recombination may serve as one of the key evolutionary forces driving the genetic diversification of AiV-D. However, the prevalence, genetic diversity, and evolutionary dynamics of AiV-D in yak populations remain largely unexplored. Given the economic importance of yaks on the Qinghai–Tibet Plateau and the potential for cross-species transmission among cohabiting livestock species, a comprehensive molecular investigation of AiV-D in yaks is warranted. The purpose of this study was to characterize the genetic features of Aichivirus D in diarrheic yaks.

## 2. Materials and Methods

### 2.1. Sample Collection

A total of 280 fresh fecal samples were collected from 280 unique yak calves with diarrhea on eight farms in Sichuan (4 farms, *n* = 216), Qinghai (2 farms, *n* = 36), and Gansu (2 farms, *n* = 28) Provinces, western China, between June 2025 and February 2026. Farms were randomly selected based on reports of diarrhea outbreaks in yak calves. Diarrhea was defined as loose or watery feces upon examination. All sampled calves were under 12 months of age. Each sample was obtained from a unique animal. Samples were transported on ice and stored at −80 °C.

### 2.2. RNA Extraction and cDNA Synthesis

In this study, total RNA was extracted from fecal samples using RNAiso Plus (Takara, Tokyo, Japan), and reverse transcription was performed using the ALL-In-One 5X RT MasterMix kit (Applied Biological Materials Inc., Richmond, BC, Canada) according to the manufacturer’s instructions. The synthesized cDNA was stored at −20 °C for subsequent analysis.

### 2.3. Screening of Yak AiV-D

The detection primer information for AiV-D was F: 5′-CTTCGCCAAACACACAACAAAG-3′; R: 5′-AAGAACAGAAGGGATAGTAGC-3′, which is 568 bp in length. After purification, the PCR product was cloned and inserted into the pMD19-T vector and subjected to Sanger sequencing (Sangon Biotech Co., Ltd., Shanghai, China).

### 2.4. Complete Genome Amplification

Among the 84 AiV-D-positive samples, four strongly positive samples from different geographic regions were selected for complete genome sequencing. Complete genome sequencing was attempted only for these four selected samples, and all four yielded complete genomes. No other AiV-D-positive samples were subjected to complete genome sequencing, and no additional samples were attempted but failed to yield complete genomes. Metagenomic sequencing, including library preparation and Illumina sequencing, was performed by Sangon Biotech Co., Ltd. (Shanghai, China) according to the company’s standard protocols. The raw data were analyzed using the CloudGetAbund platform. Briefly, raw reads were quality-filtered using Fastp v0.23.2 (Q < 15; reads with ≥40% unqualified bases or <60 bp were discarded). Host reads were removed using Bowtie2 against the Bos taurus and yak genomes. Clean reads were de novo assembled using MEGAHIT v1.2.9 (k-mer sizes: 33, 55, 77, and 99 bp). AiV-D-specific contigs were identified by BLASTn against the GenBank database and verified by Sanger sequencing. For Sanger sequencing, the genome was amplified using self-designed overlapping primer sets (Table S1). The PCR products were subsequently purified, cloned, and inserted into the pMD19-T vector, and subjected to Sanger sequencing (Sangon Biotech Co., Ltd., Shanghai, China). The resulting reads were assembled using SeqMan Pro (Version 17.0, DNASTAR, Madison, WI, USA) and ambiguous regions were resequenced for verification. In addition, four other samples with strong positive signals were selected for amplification of complete P1 gene sequences (VP0, VP3, and VP1) using the same Sanger sequencing method.

### 2.5. Genome-Wide Recombination Analysis of AiV-D

Recombination analysis of the AiV-D whole-genome sequences was performed using SimPlot 3.5.1 and RDP4 software. The AiV-D sequence obtained in this study was used as the query sequence, and the known AiV-D sequence downloaded from GenBank was used as the reference sequence. The BootScan algorithm of SimPlot (200 bp window, 20 bp step, 100 repetitions, Kimura two-parameter model) was used to detect the recombination signal. Moreover, multiple methods in the RDP4 software package (RDP, GeneConv, BootScan, MaxChi, Chimera, SiScan, and 3SEQ) were used to verify the recombination event.

### 2.6. Amplification of Complete VP0, VP3 and VP1 Sequences

The complete VP0, VP3, and VP1 gene sequences were amplified from the detected positive samples using self-designed primers. After purification, the PCR product was cloned and inserted into the pMD19-T vector and subjected to Sanger sequencing (Sangon Biotech Co., Ltd., Shanghai, China). The primer information for primers 2–6 is shown in Table S1.

### 2.7. Sequence and Phylogenetic Analysis

Genetic analysis of the identified AiV-D strain was carried out, and the gene sequence was spliced by SeqMan software and compared with the sequence in GenBank using NCBI Web BLAST (BLAST+ 2.17.0, NCBI, Bethesda, MD, USA). A phylogenetic tree was constructed using the neighbor-joining method with 1000 bootstrap replicates using MEGA12.0 software. In the family Picornaviridae, VP1 gene sequences are typically used for intraspecies genotype classification. Accordingly, in this study, genotype assignment was determined by pairwise comparisons of the complete VP1 nucleotide sequences, using a threshold of <85% nucleotide identity to define distinct genotypes.

## 3. Results

### 3.1. Detection of AiV-D in Yaks

In this study, the positive rate of AiV-D was 30.00% (84/280) in 280 fecal samples collected from yaks with diarrhea on eight farms across three western Chinese provinces (Sichuan, Qinghai, and Gansu), as confirmed by sequencing. The positive rate at the farm level was 87.5% (7/8), and seven of the eight farms tested positive for AiV-D, with positive rates of 27.78% (60/216) in Sichuan, 47.22% (17/36) in Qinghai, and 25.00% (7/28) in Gansu, indicating that AiV-D is widespread among diarrheic yak populations in these regions (Table 1).

### 3.2. Genome-Wide Feature Analysis

**Figure 1:**
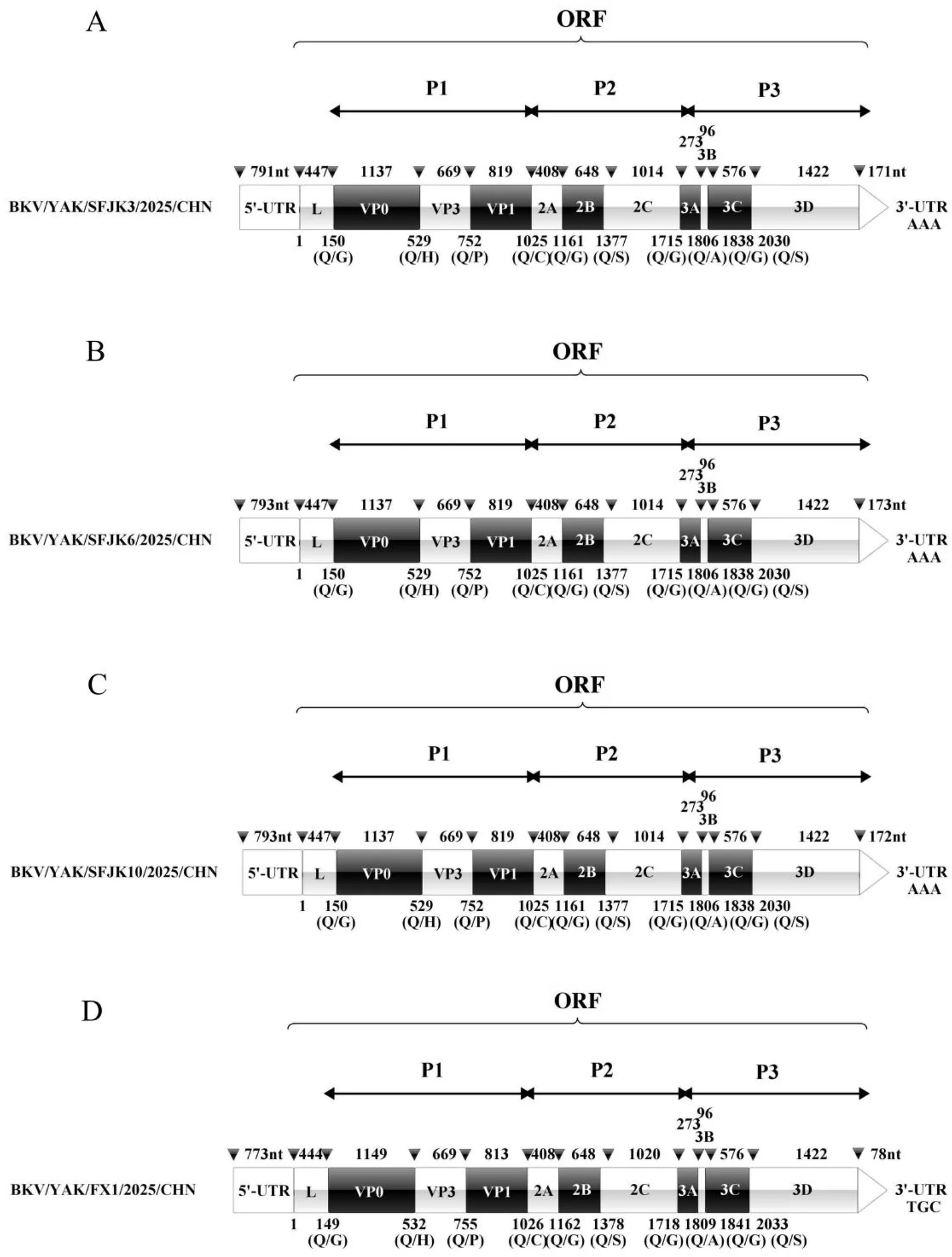
Whole-genome structure of the four yak-derived AiV-D strains. (**A**) BKV/YAK/SFJK3/2025/CHN. (**B**) BKV/YAK/SFJK6/2025/CHN. (**C**) BKV/YAK/SFJK10/2025/CHN. (**D**) BKV/YAK/FX1/2025/CHN. Each genome includes the 5′-UTR, a single open reading frame (ORF), and the 3′-UTR. The polyprotein encoded by the ORF consists of a leader protein (L) at the N-terminus, followed by three functional regions: P1 (structural proteins: VP0, VP3, and VP1), P2 (nonstructural proteins: 2A, 2B, and 2C), and P3 (nonstructural proteins: 3A, 3B, 3C, and 3D). The inverted triangle indicates the predicted cleavage site, and the amino acid switching site in the polyprotein coding region is marked below. UTR, untranslated region.

**Figure 2:**
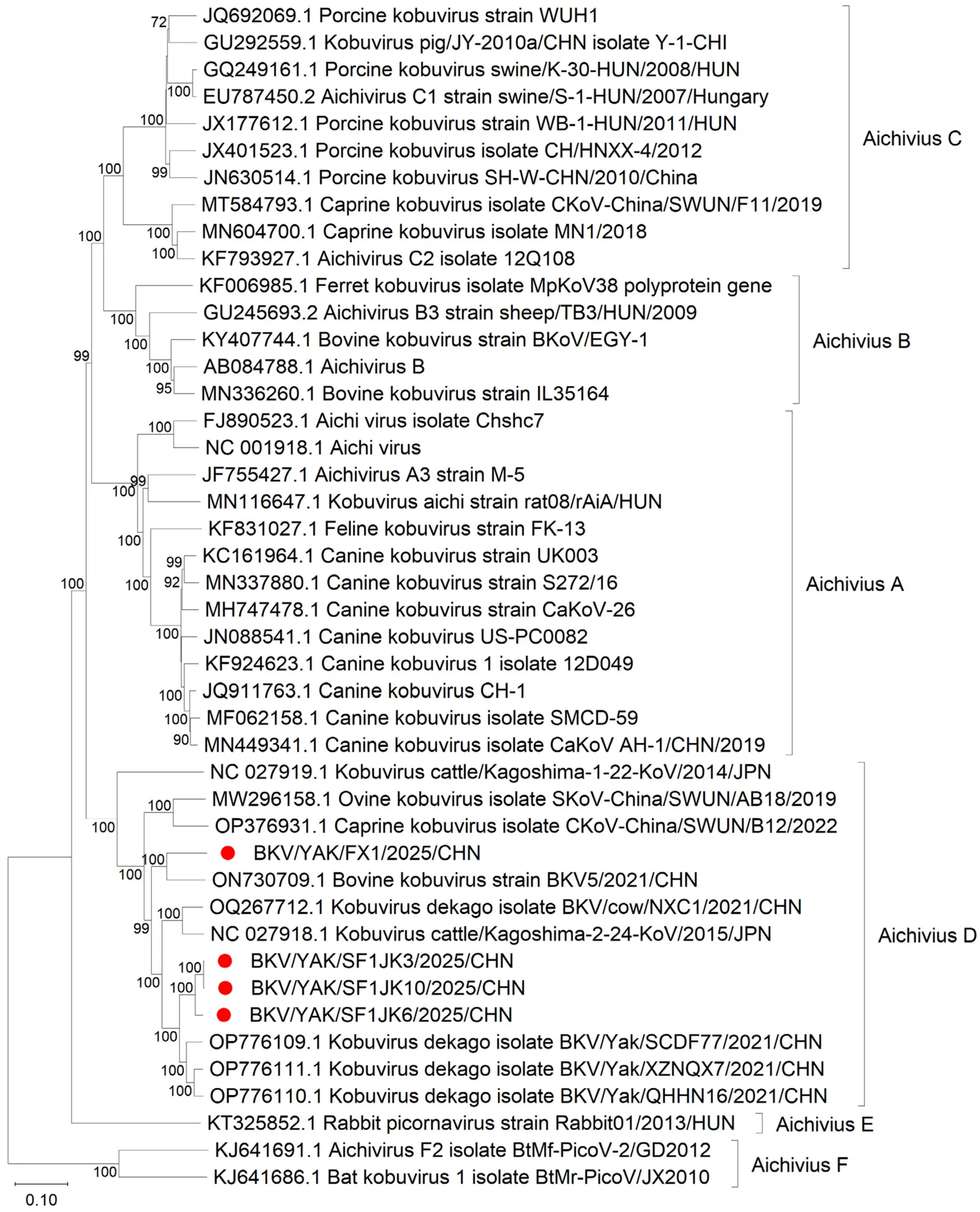
Neighbor-joining phylogenetic tree based on the complete genome sequences of all six recognized Aichivirus species (A–F). Bootstrap values (≥60%) from 1000 replicates are shown at the branch nodes. ● represents the AiV-D strain from this study.

In this study, four complete yak AiV-D genomes were obtained (GenBank: PZ424445–PZ424448) (Figure 1). The genomes ranged from 8369 to 8475 nt, with a GC content of 55.66–55.85%, and contained a single ORF (7509–7518 bp) encoding a typical picornavirus polyprotein (P1: VP0, VP3, and VP1; P2: 2A–2C; P3: 3A–3D). The ORF nucleotide and amino acid identities among the four strains ranged from 82.07% to 99.63% and from 81.96% to 99.56%, respectively. Phylogenetic analysis revealed that strain BKV/YAK/FX1/2025/CHN clustered with the bovine reference strain BKV5/2021/CHN (GenBank: ON730709.1) (84.70% nt/88.35% aa identity), whereas the other three strains clustered with the yak reference strain BKV/Yak/SCDF77/2021/CHN (89.80–93.07% nt/95.53–96.84% aa identity), all with 100% bootstrap support (Figure 2). Recombination analysis was subsequently performed to further investigate their genetic features.

### 3.3. Recombination Analysis

**Figure 3:**
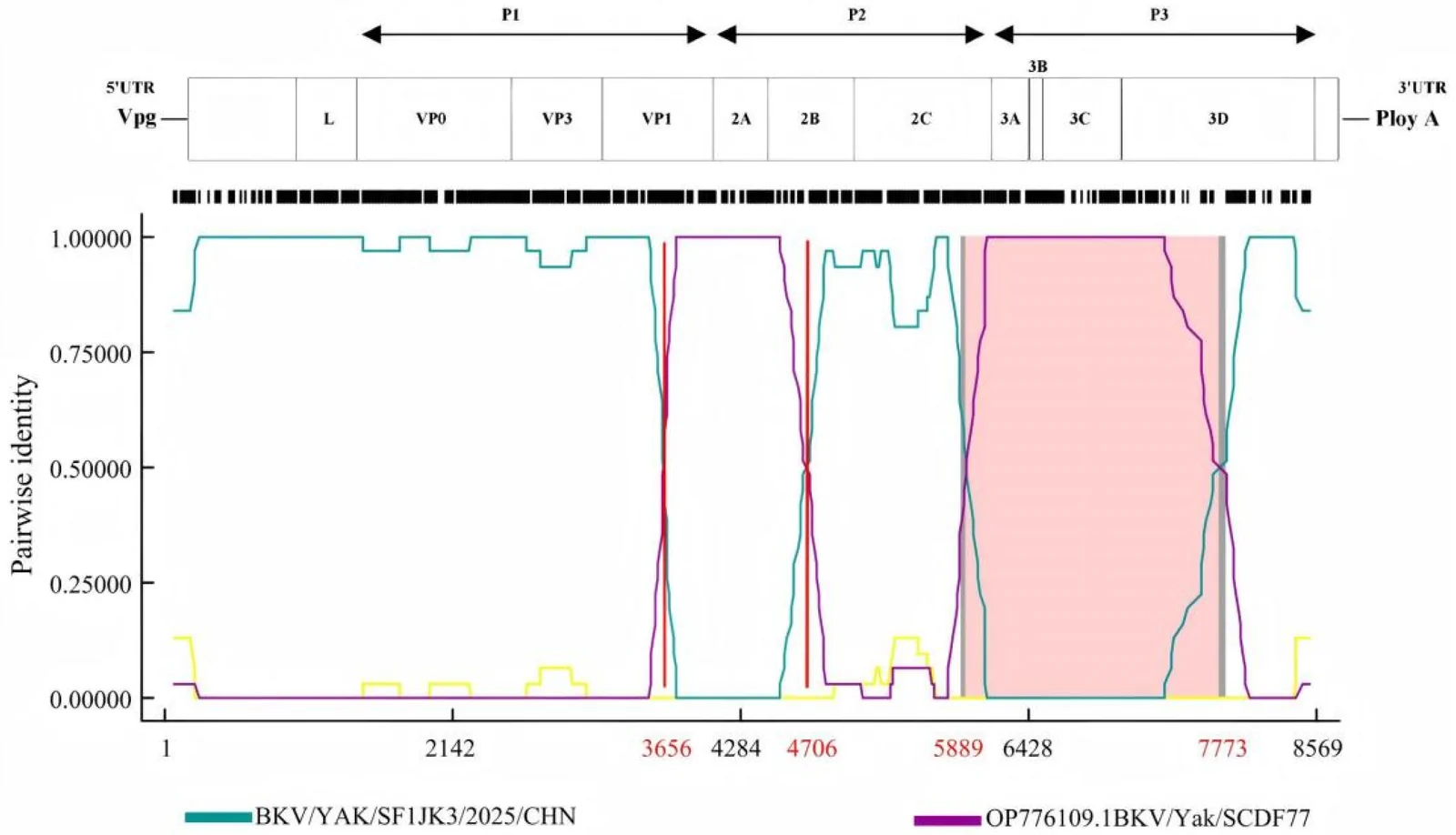
Recombination analysis of BKV/YAK/SF1JK6/2025/CHN. The genome of BKV/YAK/SF1JK6/2025/CHN was derived from the major parent BKV/YAK/SF1JK3/2025/CHN and the minor parent BKV/Yak/SCDF77/2021/CHN, with a major recombination region at nt 5889–7773 and a potential secondary region at nt 3656–4706. Recombination events were supported by seven RDP4 algorithms and SimPlot/BootScan analysis.

Recombination analysis using RDP4 and SimPlot/BootScan predicted BKV/YAK/SF1JK6/2025/CHN as a recombinant strain (Figure 3). BKV/YAK/SF1JK3/2025/CHN was identified as the major parent, and BKV/Yak/SCDF77/2021/CHN as the minor parent. A recombinant fragment spanning nt 5889–7773 originated from BKV/Yak/SCDF77/2021/CHN. An additional potential recombination signal was detected within nt 3656–4706. These predicted events were supported by seven algorithms in RDP4 and further confirmed by SimPlot. The breakpoints are located in the nonstructural protein coding region (2C–3D), representing, to our knowledge, the first report of recombination in this region of AiV-D in yaks. These findings suggest that recombination may contribute to the genetic diversity of AiV-D in yak populations, although this observation is based on a single recombinant strain and requires confirmation with additional genome sequences.

### 3.4. Complete VP0, VP3 and VP1 Sequences Obtained

**Figure 4:**
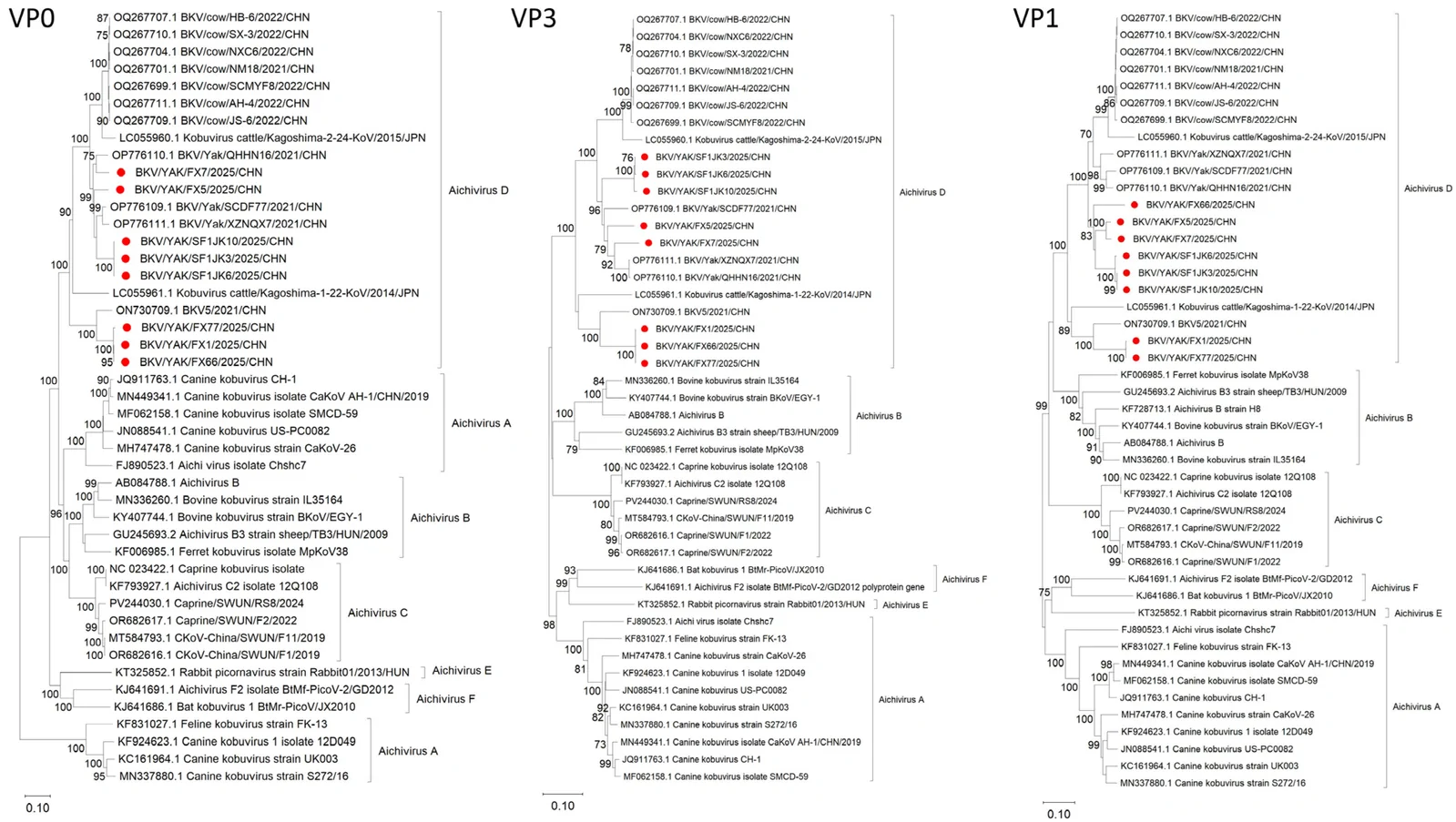
Neighbor-joining phylogenetic tree based on the complete nucleotide sequences of the AiV-D VP0, VP3, and VP1 genes. Bootstrap values (≥60%) from 1000 replicates are shown at the branch nodes. ● represents the AiV-D strains from this study.

To further characterize the P1 region, the complete VP0, VP3, and VP1 genes of AiV-D were amplified from positive samples. Eight complete P1 gene sets were obtained (GenBank: PZ424433–PZ424448), including three from Sichuan, two from Qinghai, and three from Gansu; among these, four also yielded full-length genomes. Phylogenetic trees based on VP0 and VP3 revealed that the eight strains formed two stable evolutionary clusters: five strains (BKV/YAK/SF1JK3/2025/CHN, BKV/YAK/SF1JK6/2025/CHN, BKV/YAK/SF1JK10/2025/CHN, BKV/YAK/SF1FX5/2025/CHN, and BKV/YAK/SF1FX7/2025/CHN) clustered with the yak reference BKV/Yak/SCDF77/2021/CHN but on independent branches, whereas three strains (BKV/YAK/SF1FX1/2025/CHN, BKV/YAK/SF1FX66/2025/CHN, and BKV/YAK/SF1FX77/2025/CHN) clustered with the bovine reference BKV5/2021/CHN. Notably, the VP1-based tree revealed that BKV/YAK/SF1FX66/2025/CHN shifted to the cluster containing the five yak-reference strains (Figure 4), indicating topological incongruence among capsid genes.

Sequence analysis revealed distinct amino acid substitutions between the two clusters. Compared with the yak reference BKV/Yak/SCDF77/2021/CHN, the five strains in cluster I carried four unique substitutions in VP0 (Y191N, S195T, S229D, L282F), one in VP3 (F210Y), and five in VP1 (M56V, R81Q, T85A, P149S, T199V). Compared with the bovine reference BKV5/2021/CHN, the three strains in cluster II carried 24 VP0 substitutions spanning aa 200–261, of which 11 were located within the receptor-binding domain (aa 195–253), 20 were substitutions of VP3, and 39 were substitutions of VP1, with one (D26E) located in the conserved antigenic region (aa 22–44) of VP1.

### 3.5. Genotyping Based on AiV-D VP1

**Figure 5:**
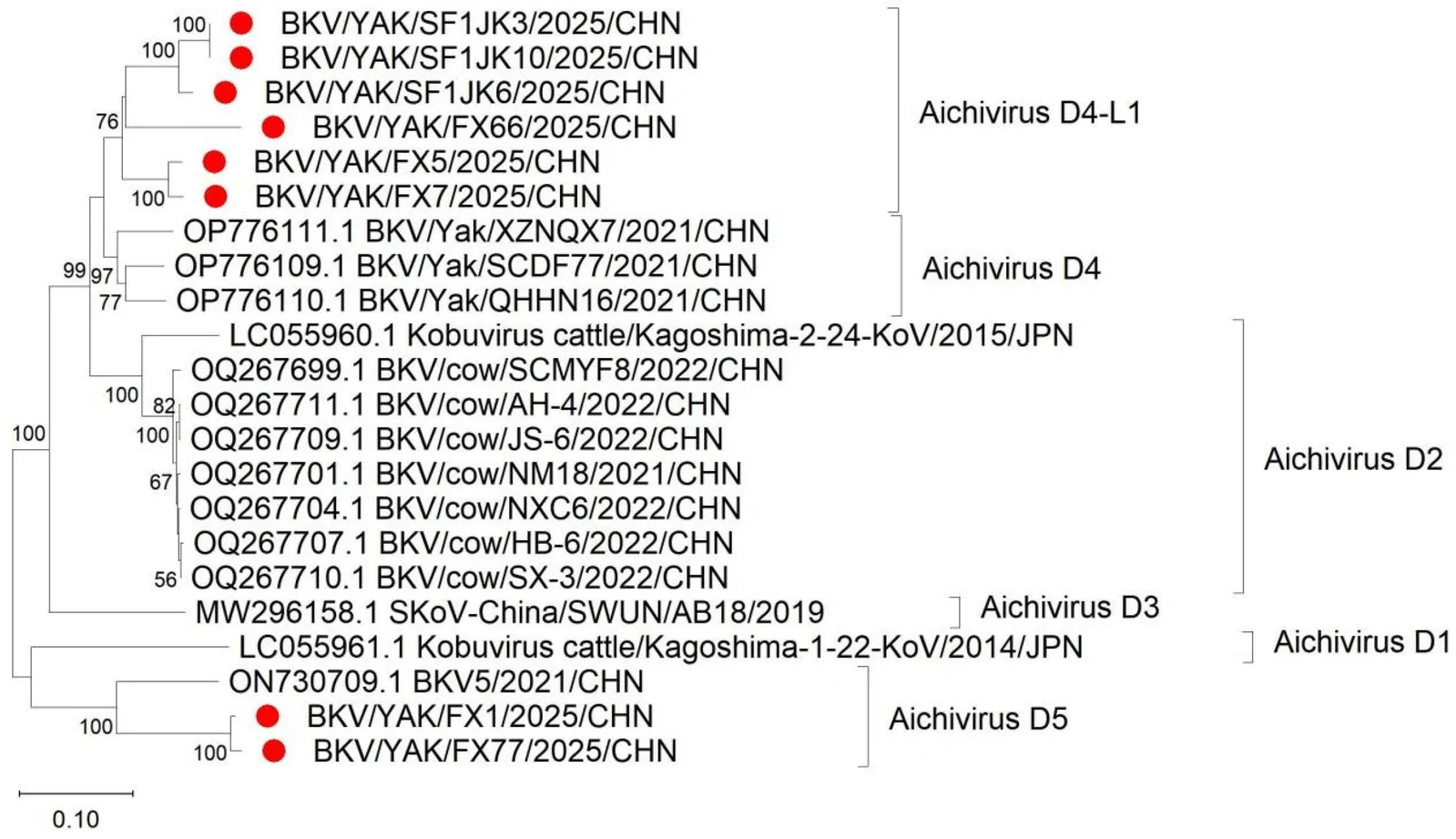
Neighbor-joining phylogenetic tree based on the complete amino acid sequences of the kobuvirus VP1 gene. Bootstrap values (≥60%) from 1000 replicates are shown at the branch nodes. ● represents the AiV-D strains obtained in this study.

Phylogenetic analysis of the VP1 gene revealed that the eight strains could be divided into two distinct clades (Figure 5): six strains (BKV/YAK/SF1JK3/2025/CHN, BKV/YAK/SF1JK6/2025/CHN, BKV/YAK/SF1JK10/2025/CHN, BKV/YAK/SF1FX5/2025/CHN, BKV/YAK/SF1FX7/2025/CHN and BKV/YAK/SF1FX66/2025/CHN) formed an independent clade and were designated as a novel sub-genotype within AiV-D4, tentatively named AiV-D4-L1, closely related to but distinct from the AiV-D4 reference strains reported in China; the remaining two strains (BKV/YAK/FX1/2025/CHN and BKV/YAK/FX77/2025/CHN) clustered with the bovine reference strain and were designated as a novel genotype, AiV-D5. These results reveal genetic diversity among yak-derived AiV-D strains and suggest possible host adaptability or cross-host transmission.

## 4. Discussion

Aichivirus D (AiV-D) is an emerging enteric pathogen belonging to the genus Kobuvirus that was first identified in cattle in Japan in 2015 [12] and subsequently detected in Tibetan sheep [13], yaks [14], dairy cattle [15], and goats [21] in China, as well as in cattle in Italy [20], suggesting a potential cross-species transmission trajectory. In this study, a relatively high prevalence of AiV-D was observed in diarrheic yaks from western China, with an overall positive rate of 30.00% (84/280) and a farm-level positive rate of 87.5% (7/8), suggesting that AiV-D is widely distributed in yak populations in this region. Compared with previous reports, the total positive rate in this study (30.00%) was higher than those reported in Japan (AiV-D1 10.4%, AiV-D2 16.9%) [12], sheep (9.2%) [13], cows (13.2%) [15], and yaks (24.8%) [14] on the Qinghai–Tibet Plateau. Regional differences were also observed: the positive rate in Sichuan (27.78%) was lower than that reported by Yan et al. (33.3%) [14] but higher than that in their other report (13.9%) [15], while the rate in Qinghai (47.22%) was higher than that previously reported (21.1%) [14]. These differences may reflect variations in host species, husbandry practices, and sampling factors. The widespread distribution of AiV-D across multiple farms in geographically distant regions suggests that the virus is widely distributed in yak populations on the Qinghai–Tibet Plateau. The genome sizes (8369–8475 nt) and GC contents (55.66–55.85%) of our strains were similar to those previously reported for yak [14], bovine [12], and dairy cattle [15] AiV-D isolates, indicating that the basic genomic organization of AiV-D is highly conserved across hosts. However, the nucleotide and amino acid identities among our four complete genomes (82.07–99.63% and 81.96–99.56%, respectively) revealed considerable genetic diversity, consistent with previous reports [13,20,21]. Phylogenetic analysis revealed that strain BKV/YAK/FX1/2025/CHN clustered with the bovine reference strain BKV5/2021/CHN, whereas the other three strains clustered with the yak reference strain BKV/Yak/SCDF77/2021/CHN, suggesting possible cross-host transmission among different livestock species. Notably, AiV-D has been detected in yaks, cattle, sheep, and goats [13,15,20,21]. Given the coexistence of these species on the Qinghai–Tibet Plateau, it is plausible that AiV-D may circulate among them. However, virus isolation was not performed in this study, and the pathogenicity of AiV-D in yaks remains to be determined. In addition to genetic diversity, recombination is another important driver of genetic variation in RNA viruses [22,23,24] and has been documented in several small RNA viruses [25,26,27,28,29,30,31,32]. Such recombination can enhance viral adaptability to new environments and influence pathogenicity [25,26,27,28]. Similar recombination-driven diversification has been documented in enterovirus 71 and other enteroviruses [33,34]. In this study, a potential recombination event was predicted in strain BKV/YAK/SF1JK6/2025/CHN. BKV/YAK/SF1JK3/2025/CHN was identified as the major parent and BKV/Yak/SCDF77/2021/CHN as the minor parent, with a recombinant fragment originating from BKV/Yak/SCDF77/2021/CHN. The recombination breakpoints identified in this study (2C–3D) are consistent with those reported in Tibetan sheep AiV-D [13] but differ from those in Italian cattle (VP1-2A junction; [20]) and goats (spanning P1 and P3; [21]), suggesting that recombination hotspots may vary across hosts. The 2C, 3A, 3C, and 3D proteins play essential roles in viral replication, host interaction, and immune evasion [35,36,37,38,39]. Therefore, recombination within the 2C–3D region may affect replication efficiency or viral fitness in yaks. To our knowledge, this is the first report of recombination in the nonstructural protein region of AiV-D in yaks, extending the known recombination landscape of this virus. A secondary recombination signal was also detected in this strain, which may indicate the possible involvement of more than two parental genomes [40]. If this recombination is confirmed, it might affect replication efficiency or viral fitness in yaks. Analysis of the P1 region further revealed genotype-specific amino acid signatures. In VP0, 11 unique substitutions were identified within the receptor-binding domain (aa 195–253) [41], a region highly conserved within the same Aichivirus species [42], potentially affecting receptor affinity or host tropism. In VP3, 21 unique changes were detected, which may influence immune modulation, as the expression of VP3 of Aichivirus C has been shown to inhibit IFN-β signaling [43]. In VP1, the receptor-binding motif (aa 227–239) [41,44] exhibited three distinct patterns among the genotypes: PRAPPTTASAPST (five strains), PRALPTTASAPLR (BKV/YAK/FX66/2025/CHN), and PRPPPSTAALLPR (BKV/YAK/FX1/2025/CHN and BKV/YAK/FX77/2025/CHN). In addition, five novel patterns were observed in the conserved antigenic region (aa 21–43) associated with neutralizing antibody production [45], suggesting potential alterations in antibody recognition and immune escape. Phylogenetic analysis based on the VP1 gene revealed a novel genotype (AiV-D5) and a novel sub-genotype within AiV-D4 (AiV-D4-L1). The D5 strains clustered with the bovine reference strain, suggesting possible cross-host circulation; however, phylogenetic relatedness alone does not demonstrate transmission between host species. Within the previously reported yak AiV-D4 reference strains, the AiV-D4-L1 strains formed an independent branch. Their presence as a distinct lineage within D4 suggests that this sub-genotype may have evolved from D4 or that they share a common evolutionary origin in the yak host. These findings further reveal the genetic diversity and evolutionary complexity of AiV-D in yak populations [16]. Owing to the limited number of farms sampled (*n* = 8) and potential within-farm clustering, province-level prevalence comparisons are presented for descriptive purposes only. Future studies with a larger number of farms should employ mixed-effects models to account for such clustering effects. These findings suggest a potential role for recombination in driving genetic diversity and highlight the need for genomic surveillance to monitor viral evolution and cross-host transmission risk.

## 5. Conclusions

In this study, a high prevalence (30.00%) of AiV-D was observed among diarrheic yaks in western China. A novel genotype (AiV-D5) and a novel sub-genotype within AiV-D4 (AiV-D4-L1) were identified. In addition, a recombination event was predicted in the nonstructural protein region (2C–3D) of AiV-D; to our knowledge, this is the first predicted report of such an event in yaks. These findings highlight the genetic diversity and evolutionary complexity of AiV-D in yaks. Enhanced surveillance is recommended for monitoring evolutionary dynamics and cross-host transmission risk.

## Figures and Tables

**Table 1:** Collection of yak feces samples and detection of AiV-D in western China.

Province	Number of Samples	Positive Rate of AiV-D (95% CI)
Sichuan	216	27.78% (22.15–34.05%, 60/216)
Qinghai	36	47.22% (31.53–63.42%, 17/36)
Gansu	28	25.00% (10.37–44.77%, 7/28)
Total	280	30.00% (24.79–35.58%, 84/280)

## Data Availability

All data generated or analyzed during this study are included in this published article and its Supplementary Information files. The GenBank accession numbers are provided in Supplementary Table S2.

## Supplementary Materials

The following supporting information can be downloaded at: https://www.mdpi.com/article/10.3390/ani16182902/s1.

## Abbreviations

AiV-D	Aichivirus D
KoV	Kobuvirus
