# Supplementary material for: Prevalence and Genomic Features of Aichivirus D in Yaks in Western China
Source: Animals (Basel). 2026 Sep 15;16(18):2902. doi: 10.3390/ani16182902 (PMC13603452; doi:10.3390/ani16182902)
Supplement: Supplementary file 1 [file animals-16-02902-s001.zip › animals-4552080-supplementary.pdf]

Table S1. Primers used for full-length genome amplification of yak AiV-D.

Primer	Sequence (5′→3′)	Fragment length
AiV-D-1F	CTCTCCTCCCACAACCCTTTTC	941
AiV-D-1R	CGGTGAGAAAGGCAAGGAGAG	
AiV-D-2F	CCACAATTGACTGCTTGACTG	1041
AiV-D-2R	GCCAGAAGAGGATTGGATAGC	
AiV-D-3F	CACCTCCAAAAAGCAAACCCCTG	988
AiV-D-3R	CAGTGTTGCCGTGCGACCATAC	
AiV-D-4F	GTTCTCTCGCCATCCTTGCCATC	1034
AiV-D-4R	CTGTTCGATGGATGAGGGGAGG	
AiV-D-5F	CACTGGTTCTGCACAGCAGTAC	1089
AiV-D-5R	GATCAGCAAAAGCAGCAGAGGTG	
AiV-D-6F	TGATACTCTCCCTCTCCTCC	844
AiV-D-6R	AGACAAAGGTGGTGCAGTT	
AiV-D-7F	GGAACAAATTTCCCTTTCCCG	911
AiV-D-7R	TGAGAGGCCTAATTTGTTGGC	
AiV-D-8F	GCGGTATTGCCACCTTCCTGAAG	893
AiV-D-8R	CCATGGGGAGAAT • GAAAGGGG	
AiV-D-9F	CCTTCGTGGTGTACCTGACTGG	1053
AiV-D-9R	GGATATTAGGCATGGCGGGTGTG	
AiV-D-10F	CCTATCACACCGTCATCCACGAC	943
AiV-D-10R	CCTCGTCCTTGAGAGAACCATTG	
AiV-D-11F	CACGGGAACAACTACACGTA	791
AiV-D-11R	CCTTCTTCACTTTTTCCAATGG	
AiV-D-12F	GATACCCCTGGAACACCATG	758
AiV-D-12R	TCAGAAGTCTTAGAGGCAGG	
AiV-D-13F	GAGATGATTGGCGGCAATCCATC	722
AiV-D-13R	CAAGTTAGGAAGAAAAGGGCGGG	

Table S2. GenBank accession numbers of the AiV-D strains obtained in this study.

Strain	Province	Sample ID	P1 genes (VP0/VP3/VP1)	Complete genome
BKV/YAK/FX1/2025/CHN	Gansu	FX1	PZ424448	PZ424448
BKV/YAK/FX77/2025/CHN	Gansu	FX77	PZ424442-44	
BKV/YAK/SF1JK3/2025/CHN	Sichuan	SF1JK3	PZ424445	PZ424445
BKV/YAK/SF1JK6/2025/CHN	Sichuan	SF1JK6	PZ424446	PZ424446
BKV/YAK/SF1JK10/2025/CHN	Gansu	SF1JK10	PZ424447	PZ424447
BKV/YAK/SF1FX5/2025/CHN	Qinghai	SF1FX5	PZ424433-35	—
BKV/YAK/SF1FX7/2025/CHN	Sichuan	SF1FX7	PZ424436-38	—
BKV/YAK/SF1FX66/2025/CHN	Gansu	SF1FX66	PZ424439-41	—
